# A clown nose‐like nodule revealing a metastatic lung carcinoma

**DOI:** 10.1002/ccr3.7249

**Published:** 2023-05-01

**Authors:** Haifa Mkhinini, Nadia Fetoui Ghariani, Sarra Yacoub, Mohamed Denguezli

**Affiliations:** ^1^ Faculty of Medicine of Sousse, Dermatology Department Farhat Hached University Hospital Sousse Tunisia; ^2^ Faculty of Medicine of Sousse, Anatomopathological Department Farhat Hached University Hospital Sousse Tunisia; ^3^ Dermatology Department, Faculty of Medicine of Sousse Farhat Hached University Hospital Sousse Tunisia

**Keywords:** clown nose‐like nodule, lung cancer, nose, skin metastases

## Abstract

Clown nose‐like lesion can be the manifestation of inflammatory, infectious or neoplasm‐related diseases, and some genetic syndromes. Lung carcinoma metastasize rarely to the skin. To our knowledge, 16 cases of lung cancer metastasis with clown nose‐like lesions have been reported. Here, we describe a new case.

**Abstract:**

Cutaneous metastases of small‐cell‐lung carcinoma are rare, and nose involvement is much rarer. However, it can be the first warning sign of lung cancer. We describe the case of a patient who presented with a red nodule of the nasal tip reminding a clown‐nose.

## Abstract 2

Cutaneous metastases of small‐cell‐lung carcinoma are rare, and nose involvement is much rarer. However, it can be the first warning sign of lung cancer. We describe the case of a patient who presented with a red nodule of the nasal tip reminding a clown‐nose.

## 1 CASE PRESENTATION

**FIGURE 1:**
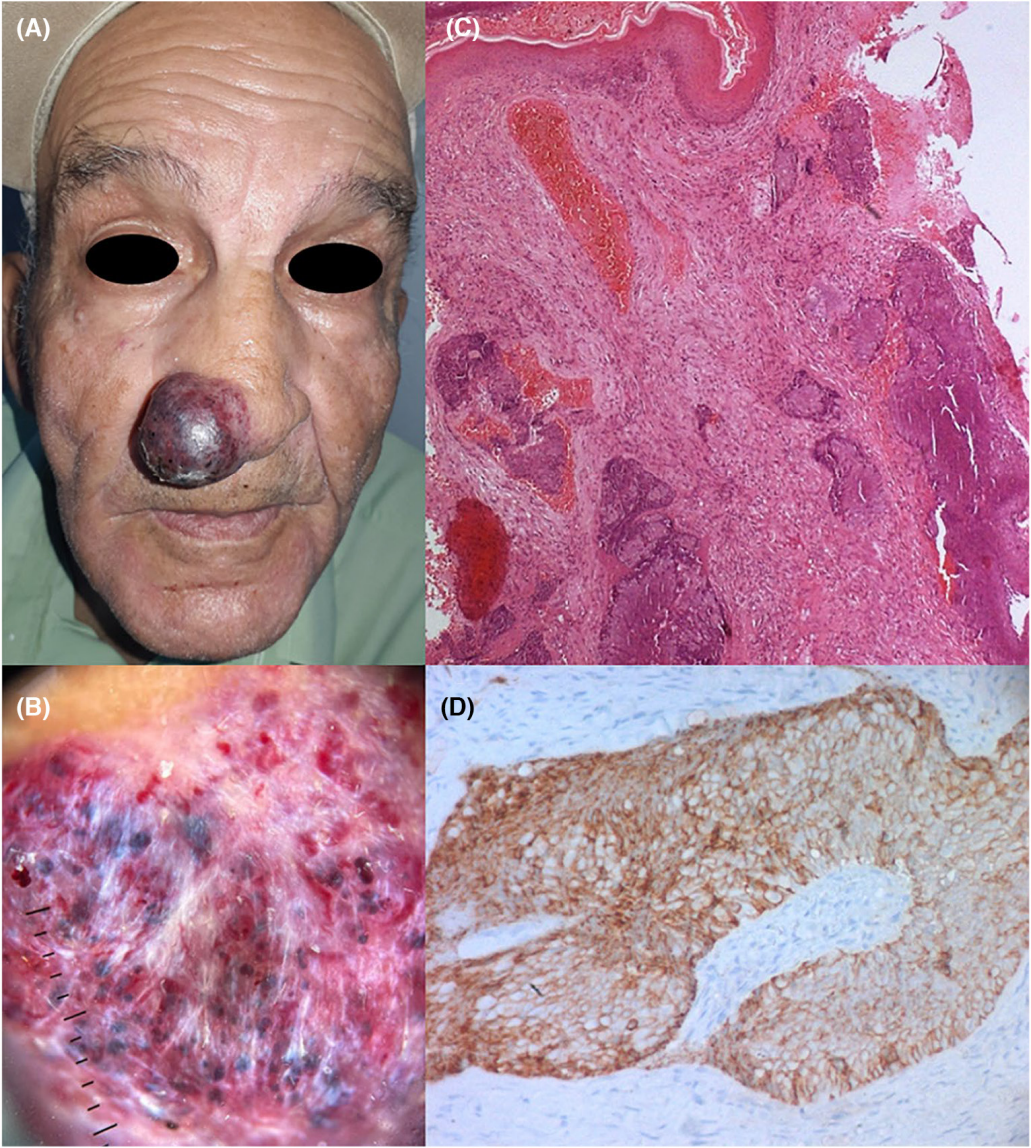
(A) Clinical picture of the red nodule of the nose. (B) Dermoscopy showed black clods and irregular vessels. (C) Microscopic examination (HEx50) showing deep infiltration of the dermis by large sheets of small blue cells with no epidermal connection. Tumor cells showed hyperchromatic nuclei with finely dispersed granular chromatin. D) Microscopic examination (x400) showing diffuse cytoplasmic immunostaining with CD56 highlighting neuroendocrine differentiation of tumor cells.

A 67‐year‐old man, heavy smoker with more than 40 pack years, presented with a nodule of the nose, evolving for 1 month (Figure 1A). He reported weight loss, anorexia, and recurrent hemoptysis for the previous 2 months. Physical examination revealed a 4‐cm‐red‐purple, angiomatous tumor located on the right wing and the tip of the nose, resulting in partial nasal obstruction. Dermoscopy showed linear irregular vessels and many clods (Figure 1B). The diagnosis of cutaneous metastasis of small‐cell‐lung carcinoma (SCLC) was made on histopathological examination (Figure 1C,D). Thoracic computed tomography scan revealed mediastinal conglomerate mass invading the right main bronchus. Our patient died 1 month later.

## 2 DISCUSSION

Small‐cell‐lung carcinoma represents 15% of lung cancers. Cutaneous metastases can occur in 1 to 12% of cases.^1^ Nasal skin involvement is extremely rare.^1^, ^2^ The onset of skin metastases has a poor prognosis^2^ with a median survival of 3 to 6 months.^2^ Cutaneous nodule with a vascular pattern on dermoscopy should orientate to skin metastases.^3^ This case confirms that a tumor of the nose can be the first symptom of an internal cancer^1^ and that dermoscopy can be a diagnostic tool.

## AUTHOR CONTRIBUTIONS

**Haifa Mkhinini:** Writing – original draft; writing – review and editing. **Nadia Ghariani Fetoui:** Supervision; validation. **Sarra Yacoub:** Formal analysis. **Mohamed Denguezli:** Supervision.

## CONFLICT OF INTEREST STATEMENT

None.

## CONSENT

Written informed consent was obtained from the patient's next of kin to publish this report in accordance with the journal's patient consent policy.

## Data Availability

Data openly available in a public repository that issues datasets with DOIs.
